# On the General Improvement in the Condition of the Insane

**Published:** 1852-10

**Authors:** 


*On the General Impro ement in the Condition of the Insane.* By M. Morel.—M. Morel observes, that since the reform of the French asylums commenced by Pinel and Esquirol, and continued by Ferrus, the physiological condition of their inhabitants has become quite changed; and remarks on their condition, which would have been just, twenty or thirty years since, are so no longer. Among the eight hundred patients of Mareville, one may seek in vain for the frightful types of degradation seen in different asylums but ten or fifteen years ago. The insane have a more civilized appearance, and are cleaner. The number of dirty patients is considerably diminished; and excrement-eaters are now only found quite exceptionally. For the purpose of clinical instruction, all the idiots and imbeciles of the asylum have been brought together, so that their special characteristics might be studied; and the graphic picture of their condition drawn by Esquirol is not now found of general application. Their condition has become ameliorated by the agency of work, exercise, gymnastics, good diet, and especially by their being transferred to healthy, well-ventilated, and well-lighted localities; for they are no longer confined within walls which prevent their view of the horizon. We must never deceive ourselves by the supposition that patients apparently in the most degraded state are insensible to the charms of nature. Surrounded by these, their features expand, and their physiognomy loses somewhat of its stupid appearance.—*Ibid.*

